# Spatial and Temporal Variability Management for All Farmers: A Cell-Size Approach to Enhance Coffee Yields and Optimize Inputs

**DOI:** 10.3390/plants14020169

**Published:** 2025-01-09

**Authors:** Eudocio Rafael Otavio da Silva, Thiago Lima da Silva, Marcelo Chan Fu Wei, Ricardo Augusto de Souza, José Paulo Molin

**Affiliations:** 1 Laboratory of Precision Agriculture (LAP), Department of Biosystems Engineering, “Luiz de Queiroz” College of Agriculture (ESALQ), University of São Paulo (USP), Piracicaba 13418-900, São Paulo, Brazil; marcelochan@usp.br (M.C.F.W.); jpmolin@usp.br (J.P.M.); 2 Laboratory of Agricultural Machinery and Precision Agriculture (LAMAP), Department of Biosystems Engineering, Luiz de Queiroz College of Agriculture, University of São Paulo, Piracicaba 13418-900, São Paulo, Brazil; thiagolim@usp.br; 3 Faculty of Civil Engineering, Architecture and Urbanism (FECFAU), State University of Campinas, Campinas 13083-970, São Paulo, Brazil; ricosouza@alumni.usp.br

**Keywords:** biennial effect, economic evaluation, field level, optimized soil fertilization, spatio-temporal analysis

## Abstract

Coffee yield exhibits plant-level variability; however, due to operational issues, especially in smaller operations, the scouting and management of coffee yields are often hindered. Thus, a cell-size approach at the field level is proposed as a simple and efficient solution to overcome these constraints. This study aimed to present the feasibility of a cell-size approach to characterize spatio-temporal coffee production based on soil and plant attributes and yield (biennial effects) and to assess strategies for enhanced soil fertilization recommendations and economic results. The spatio-temporal study was conducted using a database composed of yield and soil and plant attributes from four harvest seasons of coffee plantation in the southeast region of Brazil. We used small plots as cells, where soil, leaf, and yield samples were taken, and the average value of each variable was assigned to each cell. The results indicated that macro- and micronutrient contents in the soil and leaves exhibited spatio-temporal heterogeneity between cells, suggesting that customized coffee tree management practices could be employed. The cell-size sampling strategy identified regions of varying yield over time and associated them with their biennial effect, enabling the identification of profitable areas to direct resource and input management in subsequent seasons. This approach optimized the recommendation of potassium and phosphate fertilizers on farms, demonstrating that localized management is feasible even with low spatial resolution. The cell-size approach proved to be adequate on two coffee farms and can be applied in scenarios with limited resources for high-density sampling, especially for small- and medium-sized farms.

## 1. Introduction

Coffee is one of the world’s most traded products [1], being economically, environmentally, and socially relevant [2]. Regardless of the production system, it is necessary to improve its efficiency to support the Sustainable Development Goals [3]. At the farm level, the spatio-temporal monitoring of the variable behavior in a coffee production system can be improved, especially because it is known that yield is uneven among fields and plants [4], thus highlighting the importance of yield monitoring.

Coffee crop monitoring can be applied to several management purposes, such as (a) grain maturity [5,6,7,8], (b) diseases and pests [9,10,11,12,13], (c) biophysics aspects [4,14,15,16], (d) soil and plant nutrition [12,17,18,19,20,21], and (e) yield [4,22,23], the latter of which is also related to the biennial effect [24,25], a phenomenon that occurs every two years, represented by the yearly alternation of intensive vegetative (lower expected yield) and reproductive growth (higher expected yield).

In the literature, several approaches have been used for spatio-temporal coffee management and monitoring purposes related to high- [18,23] and low-resolution monitoring [26]. High-resolution monitoring can be considered expensive in terms of data and equipment acquisition and qualified labor requirement and is labor-intensive (e.g., maturity monitoring by workers [5]), therefore reducing the adoption rate by farmers. Thus, there is a need to develop low-cost and less labor-intensive solutions to obtain data for coffee management [27,28]. In this sense, changing plant-level management to field-level management is an alternative to overcome these hurdles.

With low-resolution monitoring as a constraint, different strategies can be applied, the management zone (MZ) approach being highlighted [29]. Researchers evaluated the application of management zones based on soil pedology from coffee farms in the southern part of the state of Minas Gerais in Brazil. Despite the possibility of applying the MZ concept to coffee farms, it is barely used because it is an approach that requires representative variables showing temporal stability in coffee farming, a perennial crop, which are not always obtainable by coffee growers.

Coffee farm plot divisions are usually relief- and logistic-related, and coffee’s grain average yield is evaluated by plot, where soil and plant management practices are conducted evenly [29]. In view of plot size, a tailored sampling strategy (cell size, grid, and stratified) [30,31] is needed according to the goal, for example, in a scenario where there is an expectation of the absence of high-spatial-resolution data for small farms, mainly due to cost. Therefore, an alternative is to apply the cell-size sampling strategy, in which the farm is divided into cells and the composite result from a sample is representative of a cell.

The cell-size sampling strategy in precision agriculture (PA) assumes the following [30,31]: (i) cell size does not necessarily have to be regular; (ii) the farmer randomly collects samples around each cell to obtain a composite sample representative of the cell, and the result of the analysis is assigned to the entire cell area; (iii) it is expected that the number of subsamples will be greater than in point sampling, as the area to be covered is much larger (the more subsamples, the higher the reliability of the value representing the cell); (iv) no interpolation is required to generate the spatial variability map of the attribute investigated. Since it is scarce to find farmers obtaining georeferenced sampling points and yield mapping for coffee production, the cell-size approach seems to be a valid, simple, and efficient strategy to be used that can improve farm variability knowledge.

Data analysis and PA tools aid the management, scouting, and investigation of the spatio-temporal aspects of soil nutrient availability, plant nutrient content, and yield. Along with the characterization of these variables, other layers of information can be obtained using PA practices, such as optimized input application and profitability maps. These practices benefit farm management, as seen in studies by Molin et al. [17] and Angnes et al. [32], because (i) they are approaches that allow the farm manager to directly guide the use of site-specific inputs, which results in adequate coffee tree yields, and (ii) they can be used to identify highly profitable and economically unviable/vulnerable areas, which can be transformed into conservation systems [33,34,35] or given appropriate directions in coffee tree management, thus improving the farm’s profitability.

Additionally, agricultural systems have been required to adapt to climate change, requiring actions that integrate agronomic, environmental, and social dimensions [36]. In this sense, e.g., for small plots within a farm, a soil sample representative of an entire small plot (cell) suggests the possibility of generating fewer samples to be sent to the laboratory when compared to other sampling methods (e.g., grid sampling). As a result, it reduces the costs, improves the adoption by the farmer, and allows for monitoring and acting on each plot differently.

Few studies in the literature have focused on understanding personalized methods of the spatio-temporal variability of soil, plant, and yield for coffee trees. In view of this, this study proposes the use of coffee small plots as cells to delving the spatio-temporal farm variability. The hypothesis of this study is that treating coffee plots as cells, using the cell sampling strategy, enhances the understanding of its spatial and temporal variability and optimizes resource use management. The general objective was to present the feasibility of applying the cell-size approach to characterize spatio-temporal coffee production. Specific objectives were to investigate the spatio-temporal yield, soil and plant macro and micronutrients among cells; evaluate the biennial effect; assess strategies for enhanced soil fertilization recommendation; and present an economic analysis.

## 2. Results and Discussion

### 2.1. Soil Attributes and Yield Spatio-Temporal Variability

**Figure 1:**
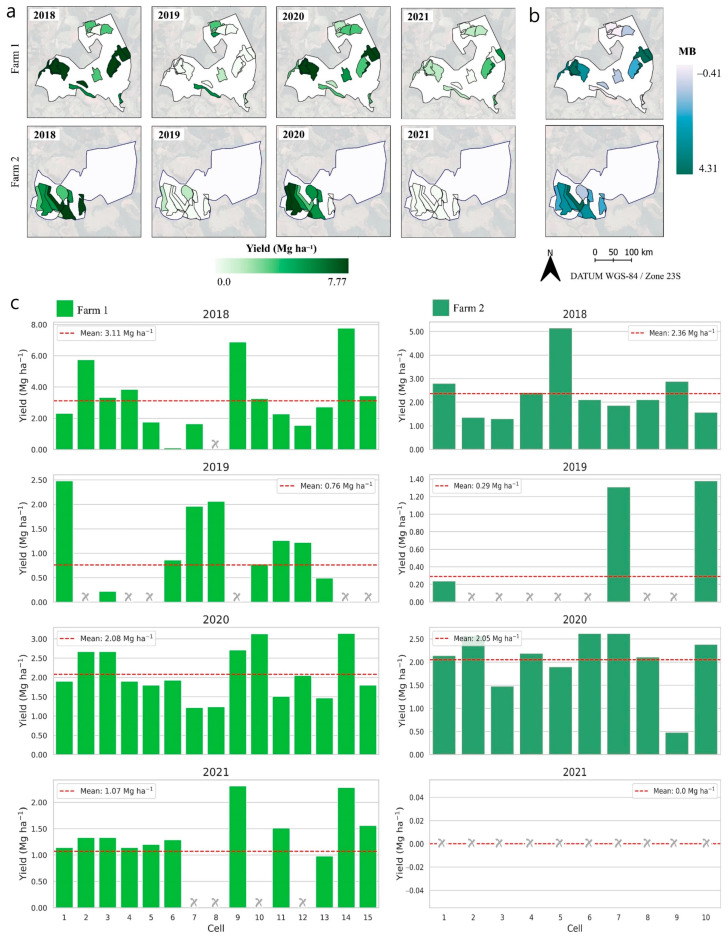
Cell-size approach for coffee yield spatio-temporal variability from 2018 to 2021. Yield by cell (**a**), spatial variability of the magnitude of bienniality (**b**), and bar charts with the yield in the cells and the average value for the farm (**c**). The dashed horizontal line in red indicates the average yield of the farm in the respective year. The x in grey indicates the yield of the coffee tree in the cell equal to zero. MB: magnitude of bienniality.

Considering 2018–2021, farm 1 presented a higher average yield than farm 2 (Figure 1a,c). In 2018, the average yield was 3.11 Mg ha^−1^ and 2.36 Mg ha^−1^ for farms 1 and 2, respectively, which is also higher than the average yield (2.01 Mg ha^−1^) of the south region of Minas Gerais in 2018 [37]. In the remaining years (2019–2021), the average yield for farm 1 was 0.76, 2.08 and 1.07 Mg ha^−1^, and for farm 2, 0.29, 2.05 and 0.00 Mg ha^−1^. These values were below the average yield for the region, on which was recorded 1.69, 2.13 and 1.31 Mg ha^−1^ for 2019, 2020 and 2021, respectively [38]. The reason for cells with no yield values recorded (0.00 Mg ha^−1^) was due to the skeletonization pruning.

Yield from both farms presented CV values ranging from 30.75% to 100%, indicating spatio-temporal cell heterogeneity between 2018 and 2021, therefore supporting the need to manage the farms unevenly and not by the mean. In this sense, yield data become an important layer to be used in conjunction with soil, plant, and weather data layers, aiding to improve the understanding of the spatio-temporal variability within the cell [22], which is of the utmost importance during low-yielding years as it can minimize the negative impacts on upcoming harvests. Spatio-temporal crop monitoring provides farmers a method to improve their data interpretation, aiding towards better management practices based on historical data. The cell-size approach demonstrates the spatio-temporal yield variability and the magnitude of the biennial effect of farms 1 and 2 from 2018 to 2021 (Figure 1).

In positive biennial years (2018 and 2020), the highest yields were 7.77 and 3.14 Mg ha^−1^ in 2018 and 2020, respectively, for farm 1. Farm 2 yielded 5.15 and 2.62 Mg ha^−1^ in 2018 and 2020, respectively (Figure 1b). These results suggest the influence of physiological recovery in 2019 and 2021, resulting in yield increment due to pruning and fertilizer applications. The negative impact of the lowest-yielding years is known in the literature and mostly related to the following: (a) imbalanced macro- and micronutrient contents in the soil and plant [27] and (b) photoassimilate allocation process in high-yielding years for grain filling. This process compromises the vegetative growth, resulting in lower yields in the next year [39]. However, in this period, the skeletonization pruning method is usually applied, causing the “zero-harvest”, contributing to the plant recovery and reduction in harvesting costs that is considered the most laborious operation, implying cost savings [40].

Understanding the physiological responses in biennial production involves interventions in the carbohydrate and nutrient cycle that influence flowering and fruit formation. Carbohydrate deficiencies are observed after high-yielding years [41], in which the plant is physiologically affected by slow growth due to decreased CO_2_ assimilation and net photosynthesis rate [42]. Some practices can contribute to the adaptation of plants during these periods, resulting in improvements in the rate of CO_2_ assimilation and conservation of the photochemical apparatus, e.g., irrigation, fertilization, artificial and natural shading, and density [43].

Beyond the biennial effect found among farms, the cell-size approach allowed us to evaluate these effects by cell. For example, in farm 1, cells were found presenting positive and negative effects, indicating different plant behavior among cells, corroborating the findings in Martello et al. [4] that relied on a higher spatial resolution (plant-level approach). These results highlight the feasibility to obtain data in supporting farmers’ decision making to improve the understanding of the biennial effect, regardless of the spatial resolution data collection.

The magnitude of the biennial effect in coffee yield can contribute to the understanding of crop behavior (Figure 1c). Values closer to zero indicate less variability, meaning a minimum difference between high- and low-productivity years. Looking at the cells, farm 1 exhibited higher biennial magnitude values, indicating greater yield variability compared to farm 2. On each farm, different biennial magnitude values are observed, suggesting intra-cell spatio-temporal variability and, as a consequence, the need for tailored management strategies at the cell level to boost crop production sustainability.

**Figure 2:**
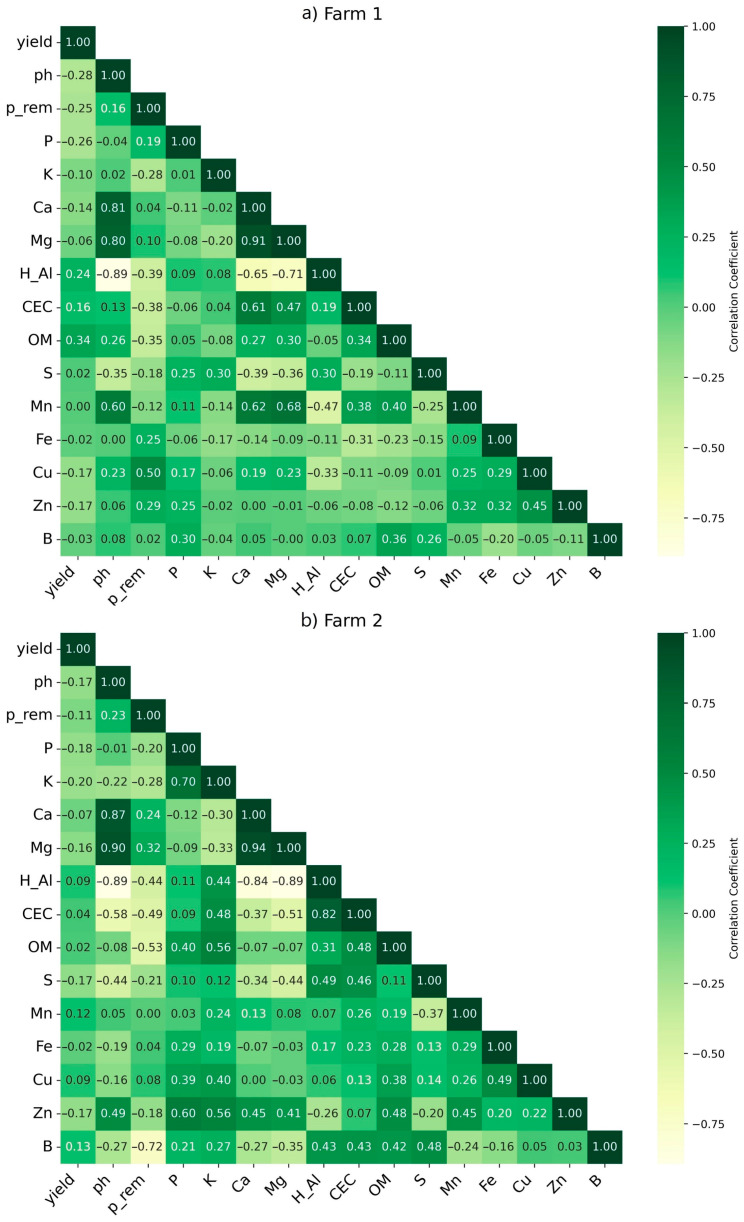
Pearson correlation matrix of soil attributes and coffee yield for farms 1 (**a**) and 2 (**b**). ph: pH using soil in water (1:2.5); P_rem: remaining phosphorus; P: Phosphorus; K: Potassium; Ca: Calcium; Mg: Magnesium; H_Al: Potential acidity; CEC: Cation Exchange Capacity; OM: Organic Matter content; S: Sulphur; Mn: Manganese; Fe: Iron; Cu: Cooper; Zn: Zinc; B: Boron.

Aiming to improve crop management, the spatio-temporal evaluation of the soil attributes related to its yield aspects is required. In this analysis, the direct correlation with the dependent variable did not consider nutrient groupings, so the standardized partial regression coefficient of the independent variable was used individually to understand its response to yield and characterize its variability [44]. Based on the correlation matrix (Figure 2), coffee yields showed positive and negative correlations with soil attributes. On farm 1 (Figure 2a), the following soil attributes showed a positive correlation with yield: Mn, S, OM and B, while on farm 2 (Figure 2b), Mn, OM, CEC, Cu and B. On the other hand, the attributes pH, P, CEC, Fe, Zn and Cu showed a negative correlation with yield for farm 1, while on farm 2, pH, P, Fe, Zn and S. From these results, it is noted that some attributes (e.g., P, P rem, CEC, Fe and Zn) were commonly related to both farms, which can be explained due to its importance to yield. For example, P is related to the growth of fruiting branches in coffee crops [20]. However, some attributes can be considered site-specific (e.g., pH and OM), highlighting that efforts towards the use of multiple data layers to improve the yield understanding is required, regardless of the spatial resolution strategies since factors such as weathering and biogeochemical cycles present a dynamic interaction among soil microbiota and other soil attributes over time and space [45].

**Figure 3:**
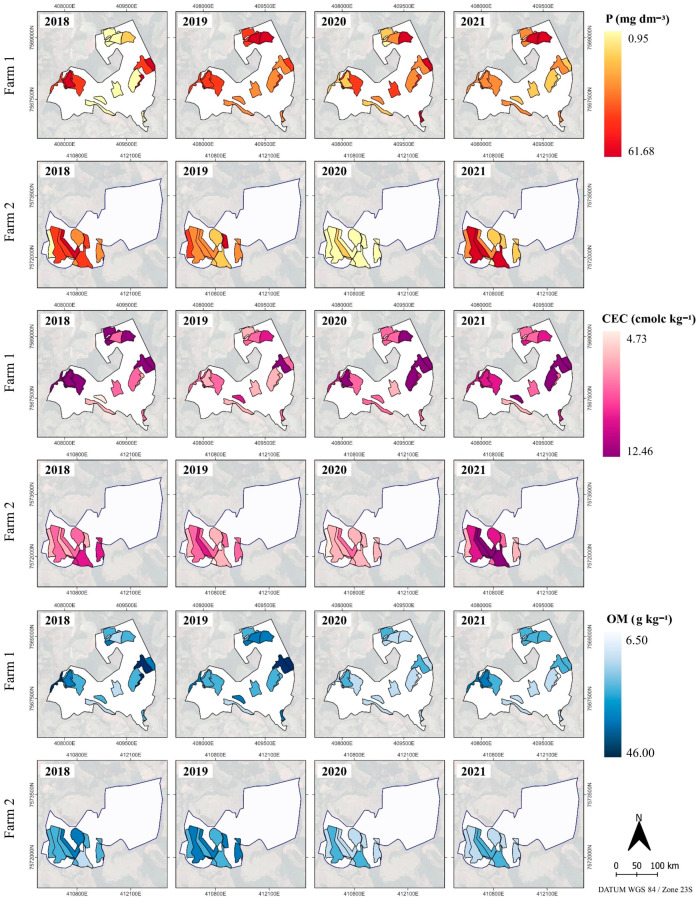
Spatio-temporal characterization of soil phosphorus (P), organic matter (OM), and cation exchange capacity (CEC) contents using the cell-size approach in coffee crops from 2018 to 2021.

Nutritional balance is considered a determining factor that affects plant growth and yield based on the excess or deficiency of certain nutrients [18,27]. Thus, the spatio-temporal variability of soil attributes must be considered (Figure 3) as specific areas of the farm (in this case, cells) because they have unique characteristics affecting the distribution and response of plants (Figure 1), as observed in the correlations (Figure 2).

The observed P contents suggest different demands for this nutrient among cells (Figure 3). For example, the third quartile of P content data for 2019 was 13.10 and 7.30 mg dm^−3^ on farms 1 and 2, respectively. However, certain cells presented maximum values of 61.68 and 16.06 mg dm^−3^ on farms 1 and 2, respectively, indicating the P spatial variability. Temporal variability can be observed comparing values between years, e.g., distinct average P values were obtained between years, with 3.01 and 3.42 mg dm^−3^ (in 2018) and 17.23 and 7.19 mg dm^−3^ (in 2019) on farms 1 and 2, respectively.

For CEC levels, a greater availability of CEC in the soil solution was identified in years with a positive biennial effect. Minimum and maximum CEC values were 5.16 and 10.89 cmol_c_ dm^−3^ (farm 1) and 5.67 and 8.35 cmol_c_ dm^−3^ (farm 2) in 2018, respectively. In 2021, the minimum and maximum values were 6.33 and 12.46 cmol_c_ dm^−3^ (farm 1) and 6.17 and 10.63 cmol_c_ dm^−3^ (farm 2), respectively. Aware that CEC is a parameter related to the Ca, Mg and K availability, its spatio-temporal monitoring is required to enhance the fertilizer application [46], in this case, monitoring with a low spatial resolution.

For OM content, the cell approach also indicated spatio-temporal variations, exposing intra- and inter-farm differences. On both farms, the largest OM amplitudes occurred in the years of 2018 and 2020. OM levels ranged from 18.70 to 46.00 g kg^−1^ (farm 1) and 18.70 to 38.40 g kg^−1^ (farm 2) in 2018, and from 6.50 to 27.40 g kg^−1^ (farm 1) and 14.10 to 26.10 g kg^−1^ (farm 2) in 2020. In 2021, the lowest OM levels were observed, possibly due to the intensified actions of cultural treatments, such as pruning and machinery traffic. Soil organic matter is one of the main components influencing quality and crop production, and its dynamics in the soil can be impacted by agricultural system activities [47,48]. Therefore, spatio-temporal monitoring in cells contributes to the understanding of intra-plot OM dynamics and to the development of strategies aimed at consciously managing land use, as well as identifying cells susceptible to degradation processes.

The variability and distribution of these nutrients observed in the soil are directly and indirectly affected by anthropogenic factors, such as fertilization and soil preparation, or environmental factors, such as topography, climate and vegetation [49]. Variability rearrangement of nutrient contents in the cells of the two farms may have been influenced by the typical terrain of coffee producing areas in the region combined with precipitation that contributes the most to K leaching, which is also affected by the microbial activities and mineral weathering. Other factors include the inappropriate use of chemical fertilizers over the years and the possibility that coffee plants are unable to make efficient use of the phosphorus available in the soil [50].

**Figure 4:**
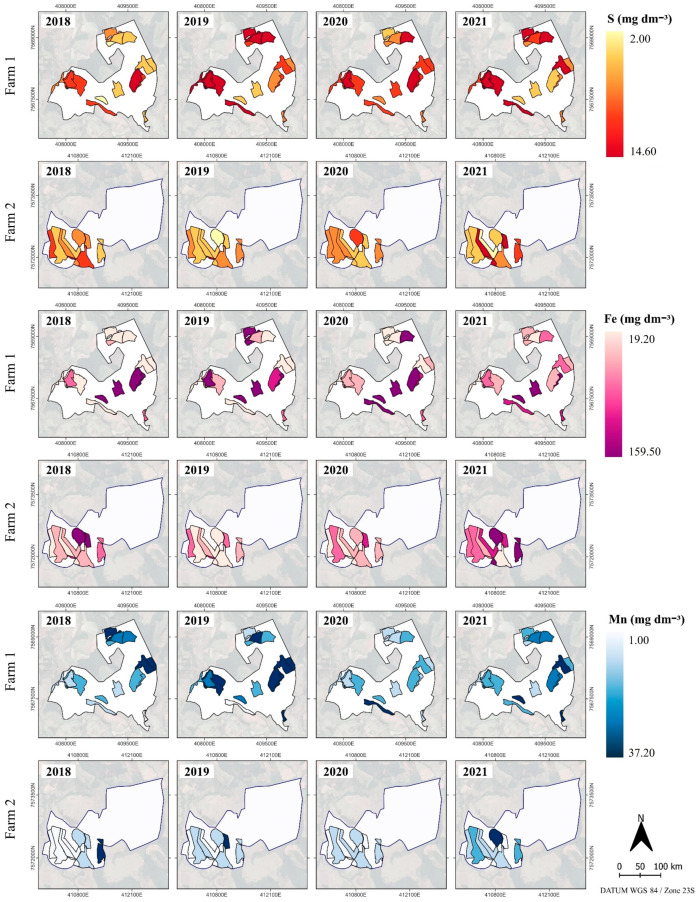
Spatio-temporal characterization of the sulfur (S), iron (Fe) and manganese (Mn) soil contents at the cell-level in coffee farms from 2018 to 2021.

In addition to P, CEC and OM, it is necessary to provide adequate amounts of other nutrients to the plant for proper development, as recommended by Liebig’s law, also known as the Law of the Minimum [51], showing the importance of constant nutrient monitoring over time and space. S distribution in the soil revealed distinct patterns over time, being more pronounced on farms 1 and 2 in 2018 and 2021, respectively. These variations are evident in the maps, where the transition from light to dark colors indicates fluctuations in S contents between cells, varying from 2.50 to 5.50 mg dm^−3^ (Figure 4).

Fe content in the soil exhibits spatial heterogeneity between cells, suggesting the need for specific and optimized fertilizer management on farms at the cell level. Spatio-temporal variations were also observed for Mn, B, Zn and Cu. The Mn spatial distribution between cells indicated an increase in lower yield years. In certain cells on farms 1 and 2, there was a low temporal variation of Mn, with levels below 7.00 and values above 22.00 mg dm^−3^ over the years. The highest B and Zn contents in the cells of farms 1 and 2 coincided with low-yielding years.

The importance of these micronutrients for coffee plant development is known: (i) Fe affects processes, such as nitrification, respiration and synthesis of genetic materials [20]; (ii) Cu has a positive effect on grain yield and contributes to the protection mechanism against diseases and to a reduction in leaves’ ethylene production [18]; and (iii) Mn assists in the photochemical release of O_2_ in the Hill reaction of plant photosynthesis [18].

### 2.2. Spatio Characterization of Coffee Leaf Nutrient Content

**Figure 5:**
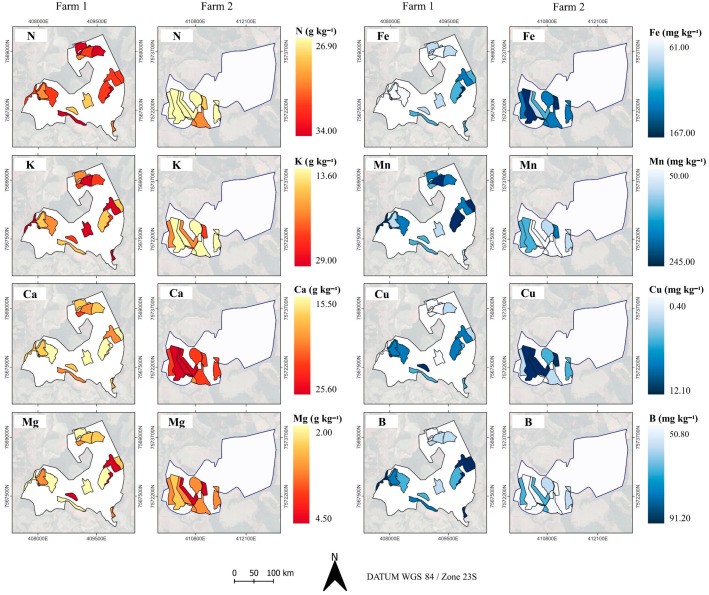
Spatial variability of leaf analysis (Nitrogen—N, Potassium—P, Calcium—Ca, Magnesium—Mg, Iron—Fe, Manganese—Mn, Copper—Cu, Boron—B) in 2020 at the cell-level in the coffee farms.

The spatial distribution of coffee yield (Figure 1a) compared with the macro- and micronutrient contents of the plant leaf, especially N, Ca, Mg, Cu and Mn (Figure 5), is a valuable method to look for patterns. For example, it can be seen that cells with higher levels of these nutrients presented a higher coffee yield in 2020. For the leaf nitrogen content, there was greater spatial heterogeneity of values for farm 1 compared to farm 2. N is the most required element by *Coffea arabica* as it plays a fundamental role in the formation of amino acids, proteins, chlorophyll and enzymes essential for photosynthesis. Its deficiency results in a severe reduction in plant development [52]. Considering N content and historical yield data at the cell level, improved N fertilizer recommendations can be recommended, meeting the plants’ nutrient requirement, providing several benefits, such as reduced N runoff and leaching into groundwater [53].

Ca, Fe, K and Mg spatial variability obtained from leaf analysis suggests that cells can be treated unevenly because of the heterogeneity found (Figure 5). These nutrients play different roles in the plant development. Ca influences coffee tree growth, yield and stress control [20]; K impacts coffee grain yield (low concentration limits yield potential and high concentration impairs Ca and Mg absorption [27]); and Fe relates to the plant growth and can be affected by the imbalance of Cu, Mn and Zn through competitive inhibition [18]. Leaf analysis at the cell level provided a method to evaluate the spatial variability of the nutritional levels on the plants, which are related to the vegetative growth, fruit ripening and yield. Thus, even applying a low-spatial-resolution sampling strategy, it is possible to visualize heterogeneity among cells, indicating that management can be improved applying customized solutions by cells within the farm, instead of using the average management throughout the farm.

### 2.3. Optimizing Fertilization in Cell-Size Resolution

**Figure 6:**
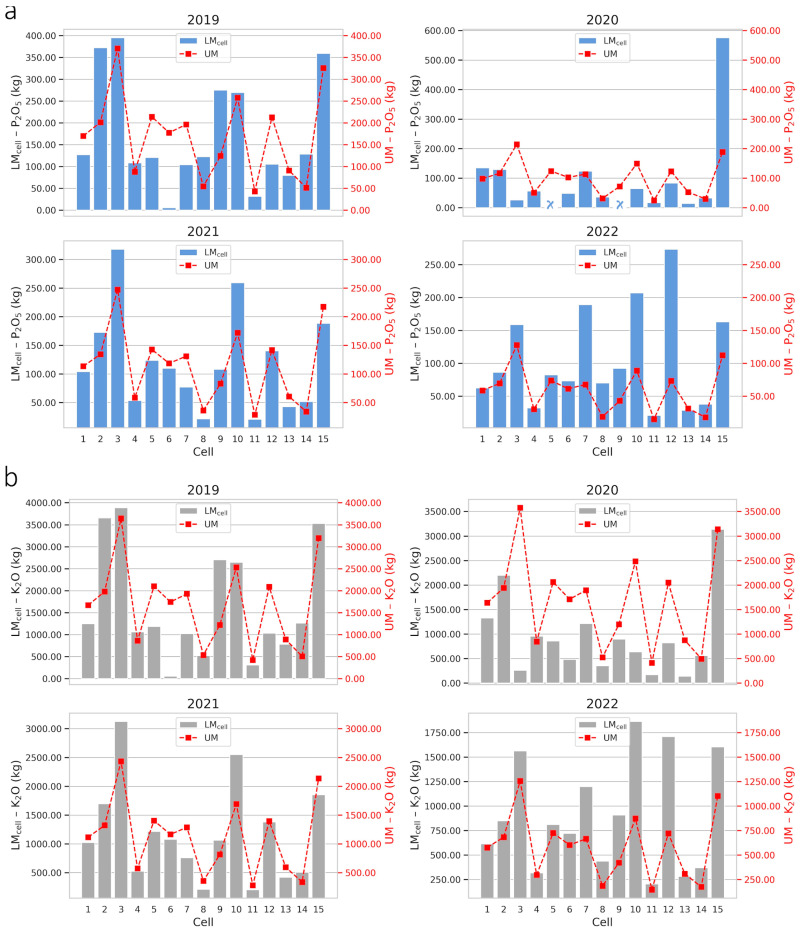
Total recommendations for phosphate (P_2_O_5_) (**a**) and potassium (K_2_O) (**b**) fertilizers by the uniform management (UM) and localized management in cell-size resolution (LM_cell_) strategies for farm 1 for 2019 to 2022. Red squares and x blue on the axis corresponding to zero indicate that the recommendation is not to fertilize using the UM and LM_cell_ strategies, respectively.

**Figure 7:**
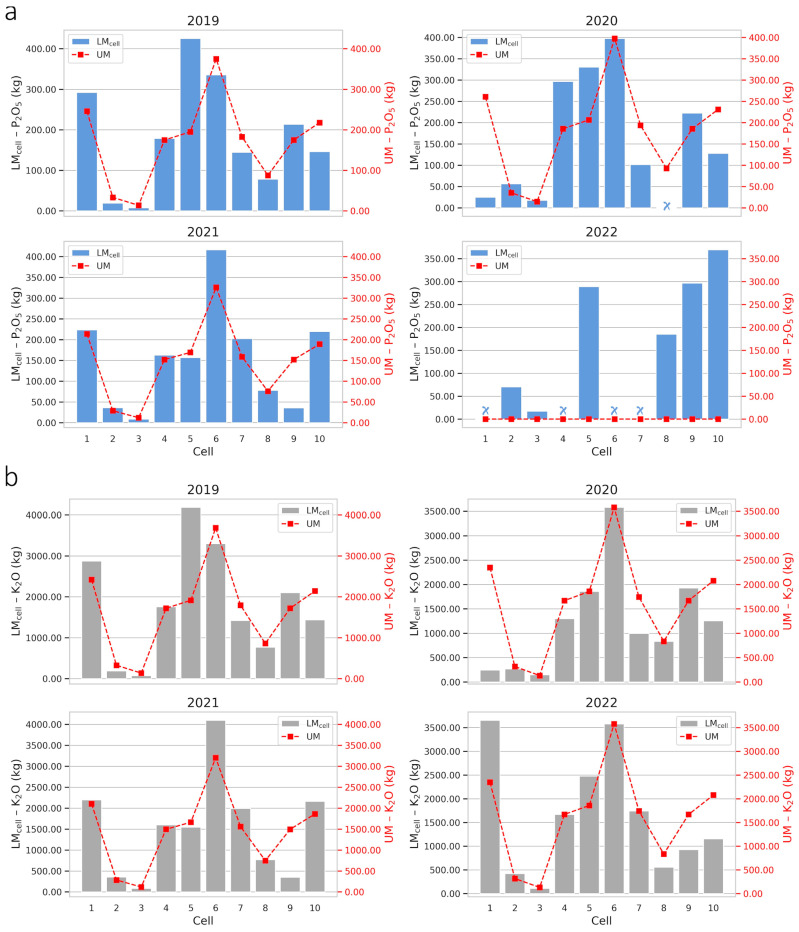
Fertilizer recommendation strategies for farm 2. Total recommendations for phosphate (P_2_O_5_) (**a**) and potassium (K_2_O) (**b**) fertilizers by the uniform management (UM) and localized management in cell-size resolution (LM_cell_) strategies for farm 2 for 2019 to 2022. Red squares and x blue on the axis corresponding to zero indicate that the recommendation is not to fertilize using the UM and LM_cell_ strategies, respectively.

P_2_O_5_ and K_2_O fertilization recommendations using the UM and LM_cell_ methods were different and showed variability between cells and farms. Considering the LM_cell_, around 46.67% and 38.75% of the recommended doses of P_2_O_5_ and K_2_O on farms 1 and 2, respectively, were lower than the recommendations based on the UM, for the period from 2019 to 2022. Most of the cells showed a higher demand for nutrients in the soil by LM_cell,_ indicating that, depending on the cell, UM overestimated the required dose, which is reflected in the total levels recommended for each cell in their respective years (Figure 6 and Figure 7). P_2_O_5_ and K_2_O doses, in kg per hectare, for the UM and LM_cell_ strategies on farms 1 and 2 are available in Supplementary Material S1 (Figures S1 and S2).

Total fertilizer recommendations using the UM strategy exceeded the LM_cell_ values for K_2_O on farm 1 in 2019 (Figure 6a) and for P_2_O_5_ and K_2_O on farms 1 (Figure 6a,b) and 2 (Figure 7a,b) in 2020, indicating a surplus of inputs. However, coffee growers and decision makers must be careful when interpreting these results, because at the cell resolution, it was found that some cells showed a surplus, while in others, the recommendation per UM was lower than per LM_cell_. Therefore, the recommendation of phosphate and potassium fertilization according to the average nutrient levels on the farm does not rationally meet the local nutritional needs and, for this reason, the LM_cell_ fertilizer recommendation is the appropriate one. P_2_O_5_ recommendation for 2022 on farm 2 is an example of the optimized use of inputs, where the UM strategy indicated no fertilization for that year, while the LM_cell_ recommended fertilizer applications in certain cells. Thus, using PA practices will not always indicate a reduction in the use of inputs but, rather, a more assertive recommendation about the local nutritional demand, ergo a more efficient use.

Fertilization recommendations differ because the traditional method uses the average yield of the farm as the basis for calculation, while the cell-size method considers the specific yield of each cell. In “zero-harvest” years, both methods rely on soil analysis, but the traditional method uses the farm’s production history, whereas the cell-based method focuses on the history of the specific cell, optimizing the process. This localized approach of the cell method generates more accurate recommendations compared to the traditional method (global data).

Table 1 shows total P_2_O_5_ and K_2_O recommended for application on farms 1 and 2 from 2019 to 2022. Regardless of the the scenarios, the recommended destination of fertilizers in the cells is different between the UM and LM_cell_ strategies, since LM_cell_ only directs the necessary inputs to the cell. For farm 2, for example, the percentage difference between UM and LM_cell_ in the K_2_O recommendation in 2022 is close to zero, indicating similar total quantities of K_2_O on the farm, but the fertilizations recommended in each cell are different for UM and LM_cell_. This targeting of inputs for each cell is relevant mainly because adequate potassium fertilization has the potential to improve the physical, chemical and sensory attributes of coffee grains [54].

Although in most cases, the LM_Cell_ strategy requires a greater amount of fertilizer, it provides an optimized application, boosting yield and reducing losses due to the surplus or deficiency of nutrients in the soil. For instance, on farms 1 and 2, P_2_O_5_ recommendations increased by an average of 18.83% and 25.00%, respectively, for the cell strategy (LM_cell_) compared to the UM method. For K_2_O, recommendations increased by an average of 3.31% (on farm 1) and decreased by 2.63% (on farm 2) for the LM_cell_ compared to the UM over the four years of study. Although fertilizer costs rise due to this additional demand, the more accurate distribution contributes to increased yield, reducing the occurrence of undernourished areas that could compromise yield. When considering the average cost of fertilizers and the value of additional yield in a high-value-added crop, the LM_Cell_ strategy can turn this investment in fertilizers into significant economic returns, increasing the quantity and quality of coffee harvested.

Other studies have evaluated the optimization of P_2_O_5_ and K_2_O in coffee crops. Angnes et al. [32] evaluated fixed-rate (uniform) and variable-rate (localized) applications of these fertilizers in two coffee crops, using recommendation maps of these inputs generated from grid sampling and the kriging spatial interpolation method. They found savings in fertilizer management at variable rate without compromising yield. Molin et al. [17] also found favorable results for coffee tree yield with the use of PA practices for the application of potassium and phosphate fertilizer compared to uniform application, with savings in inputs for phosphate fertilizer and increased use of potassium fertilizer in localized management. Therefore, even with different soil sampling strategies and spatial resolution of the coffee plantation, the studies in the literature corroborate the results observed for the approach considering local application at cell resolution. This indicates that this approach is a way of optimizing the use of inputs in a rational manner aligned with the goals of a more sustainable production [3].

### 2.4. Profitability Maps at the Cell Level

**Figure 8:**
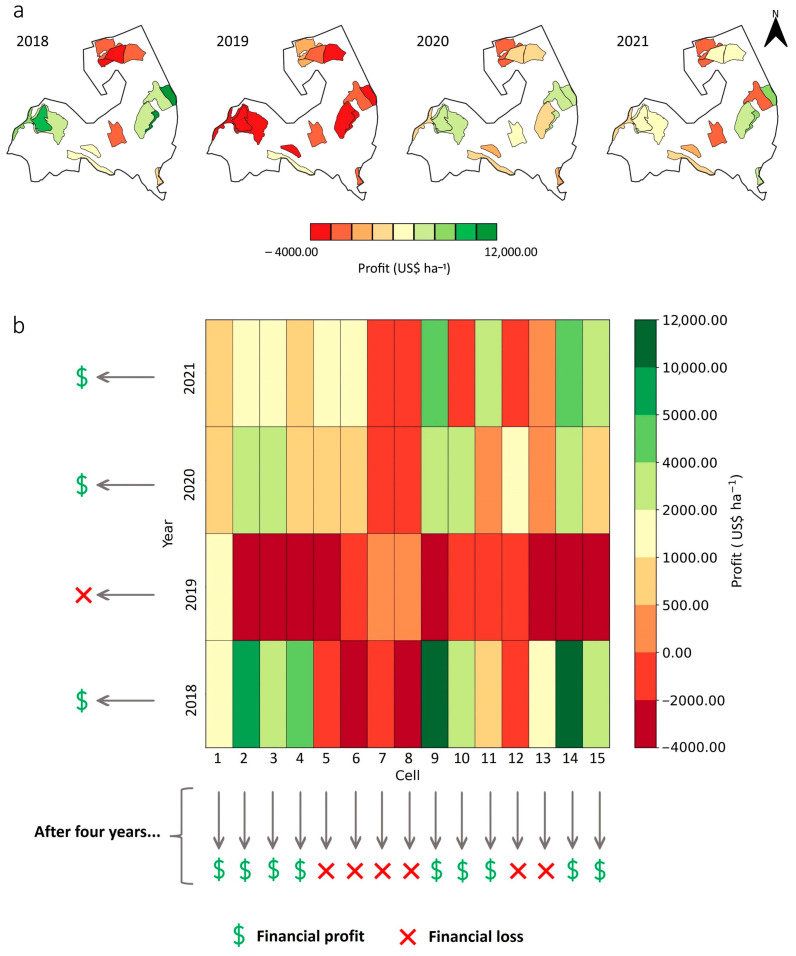
Spatial characterization (**a**) and heat map (**b**) of the profitability of coffee production on farm 1 for 2018 to 2021.

In 2018, cells with profitability above USD 10,000.00 ha^−1^ and bellow USD −2000.00 ha^−1^ were identified (Figure 8). The good performance of coffee production in the Minas Gerais region in 2018, the largest producer of *Coffea arabica* in Brazil, was attributed to favorable climatic conditions, vigorous flowering, adequate cultural treatments and drought during the harvest period that provided uniform fruit growth and ripening [37]. Further, 2020 presented the highest number of cells with positive profitability values compared to the other years, while, in 2021, a greater number of cells showed negative profitability, mainly related to the physiological effects related to the negative biennial year, favoring the post-flowering abortion and consequent yield loss [38]. In fact, there are countless factors that affect crop profitability, including climatic issues, plant physiology, management and volatility of costs related to external factors, such as the exchange rate of the US dollar (USD) against the Brazilian real (BRL), which has increased over the years.

Certain cells showed a negative biennial effect, even though the cycle was characterized as a positive-yielding year. On farm 1, this occurred in 2018 for one cell, where no yield was obtained. Furthermore, it was observed that yield data equal to zero were recorded in some cells in 2019 and 2021 (negative years) due to the absence of harvest in these cells because of the “zero-harvest” system (Figure 8). Therefore, there was no income associated within these cells; however, there were costs to maintain the crop, e.g., pruning system and leaf and soil fertilization, which ranged from USD 121.28 ha^−1^ to USD 2971.45 ha^−1^. In general, profitability maps highlight the variability in the economic performance of coffee farms. By analyzing these maps at the cell level, the farmer can tailor site-specific management strategies for different purposes such as enhanced fertilizer use efficiency and cultural treatments aiming to minimize production costs and improve economic net return [55].

Figure 8b shows the profitability of coffee production, indicating positive and negative profit through the transition between green and red tones. Looking at the total of the cells for each year, in the horizontal direction, it can be seen that all the years studied showed a financial gain, except for 2019, which showed negative profitability values. It was observed that there were cells that showed a financial loss, even though the total profitability of the farm indicated a financial gain for that year. For example, the total profitability of the farm in 2018 indicated a financial profit; however, when analyzing the cells individually, it was found that five cells presented financial losses (cells 5, 6, 7, 8 and 12).

The “zero-harvest” system occurred in cell 8 in 2018, which justifies its financial loss, given that it is an investment in inputs and management aimed at adequate production for the following year and expected return on this investment. However, for the remaining cells, it is an indication that the farmer paid to produce the coffee, which is not expected in an agricultural enterprise. Therefore, if not investigated individually (by cell), the analysis of financial profit or loss can be misleading and, over time, can even lead to the interruption of coffee cultivation due to failures in management and interpretation of the results.

In 2019, even in the absence of harvesting six cells due to the “zero-harvest” system (cells 2, 4, 5, 9, 14 and 15), there were cells harvested that presented low yield values (cells 3, 6, 10 and 13). As there were costs related to this operation, the cost of producing coffee increased in these cells, exceeding the profitability of that year’s production and consequently resulting in a financial loss. Harvesting operation accounted for around 20.00% of the effective operating production cost in each year, making it one of the most costly operations. This underscores the importance of harvesting coffee at its maximum production potential and quality.

Observing Figure 8b vertically, it is possible to see the profitability per hectare of each cell over the years. When analyzing profitability over time, six cells had total revenues lower than their costs, indicating financial losses for the farmer (cells 5, 6, 7, 8, 12 and 13). At the end of the four-year study, nine of the fifteen cells showed financial gains on farm 1. In cells 7 and 8 in 2018, 2020 and 2021, for example, the revenues obtained from the activities carried out were not able to generate profit, as they did not cover production costs. In cells 5, 6 and 13, there was a profit in 2018, followed by a loss in 2019 and a profit again in 2020 and 2021. It is a fact that decision making in coffee cultivation based on profit/loss must consider historical data, since, depending on the expression of profitability in the cell, these values are diluted over the years.

Obtaining coffee plantation profitability by total production on the farm, without considering the location and distribution of this production in each cell, may not be the most appropriate approach in achieving a positive economic return. The spatio-temporal analysis of profitability guides appropriate management, such as the decision to invest in technologies and inputs in certain locations on the farm, scheduling of pruning operations [56,57] synchronized with years of negative bienniality, decisions to reform the plantation and the transformation of economically unprofitable cells into conservation areas [34], with the potential to balance economic, ecological and social interests.

The hypothesis that small coffee plots can be treated as cells by using cell sampling and management approaches to enhance the spatio-temporal variability of the farm in terms of fertilizer recommendation and profitability was confirmed in this study. The results from this study indicate that decision making related to the coffee agricultural management can be improved using multiple data layers (yield, soil and plant macro- and micronutrients and profitability) considering the cell-level approach, a low spatial but feasible resolution strategy. Furthermore, this approach can be extended beyond the two farms tested. In this sense, it is shown that coffee crop production can be more sustainable without being limited to the use of high-resolution strategies, therefore being suitable for any size of farmers, especially those that have restricted access to high-resolution tools.

Beyond obtaining cell profitability, complementary analyses must be adopted to facilitate farmers’ decision making, including the assessment of climate risk and fluctuations in commodity prices [58], access to capital credit, reduction in labor costs [59], and political scenarios that contribute to changes in input prices. The use of information together must be adopted for assertiveness in agricultural activities, reducing risks and creating empirical models to guide operations. The comparison between management methods must be carefully analyzed, considering economic, social and environmental factors as predictive attributes in decision making to foster sustainable agricultural production.

### 2.5. Advantages, Limitations and Futures Studies

The approach of this study proposes a solution to the limitations in monitoring and obtaining data with PA practices in coffee production. This method was introduced in the literature in the 1990s [30], in the 20th century, but it still requires broader dissemination, especially among small- and medium-sized farmers. Its application has the potential to enable PA and farm management for all producers. In this context, the described approach proves to be a strategy that does not demand complexity for optimizing inputs, something essential in agricultural systems for small-scale farmers, where crop yield is often limited by the availability of nutrients such as P and N [60]. Furthermore, the approach contributes to sustainability and increases the nutritional value of the crop, positively affecting food security.

The presented results highlight several advantages of the cell-size-based approach compared to other crop management methods: (a) It is a lower-density and less complex approach compared to georeferenced sampling methods, such as targeted sampling and grid sampling [61,62]. In cell-size sampling, a high density of subsampling is performed, generating only one composite sample per cell, whereas georeferenced methods require higher densities of sampling and subsampling within the plot, making them more costly and time-consuming. (b) By generating a representative sample of the cell, spatial and temporal analysis is simplified, eliminating the need for complex analyses such as geostatistics, interpolations and advanced processing for the creation of management zones. These processes often require dedicated software and specialized computational skills, which can limit their use in scenarios with less access to technology and training. The cell-size approach presents less mapping complexity for agronomic decisions. Geostatistical methods require the integration of several maps into a single one, in a rational way, considering agronomic and practical reasons [12]. When generating management zones, synthesizing chemical and physical variables for homogeneous management is challenging due to high variability, which affects the definition of appropriate zoning. In addition, it is necessary to assess the stability of spatial variability in the plot, as temporal variability tends to be greater than spatial variability [12].

Studies in the literature, such as that by Valente et al. [63], demonstrated the efficiency and accuracy of cell sampling for low-density sampling scenarios compared to other methods. This study evaluated various sampling techniques for mapping soil attributes, including high- and low-density point grid sampling, cell sampling, management zone sampling and the conventional method (average). The cell sampling method, with cell sizes ranging from 0.40 to 12.60 ha, showed the lowest root mean square error (RMSE) for most soil attributes analyzed, and it was comparable to other soil attribute mapping methods.

Cell sampling, while offering advantages such as saving resources compared to other forms of sampling, has some limitations: (i) it is an approach that is penalized in terms of spatial resolution and detail, which hinders the identification of plant-level variability making. This makes it difficult to monitor the coffee tree’s phenological cycle in detail at an individualized resolution; (ii) with the method proposed in this study, there is less room for error in decision making (e.g., managing inputs and profits), but there are still more errors inherent in using cells than other higher-spatial-resolution approaches.

For future studies, the use of a control group should be considered to directly compare differences in coffee yield, changes in soil fertility and economic benefits between the use of the cell-size approach and traditional management methods. Additionally, other data layers from satellite images (e.g., vegetative indices, altitude, and climatological data) could be incorporated; this could improve monitoring and provide additional support for decision making in coffee crop management.

## 3. Materials and Methods

### 3.1. Data Collection

**Figure 9:**
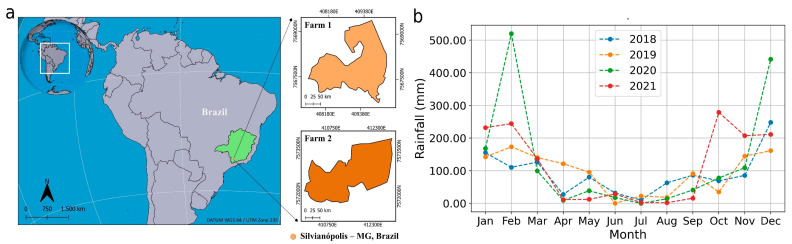
Geographic location of farms 1 and 2, municipality of Silvianópolis, Southern Minas Gerais State, Brazil (**a**) and monthly rainfall from 2018 to 2021 (**b**).

This study was carried out on two commercial farms cultivated with *Coffea arabica* L. located in the municipality of Silvianópolis, Southern Minas Gerais State, Brazil (Figure 9a). According to the Köppen classification, the climate is Cwa (monsoon-influenced humid subtropical climate) with moderate temperatures, warm and rainy summers, visualized from historical monthly rainfall data in the study region (Figure 9b). Soils were classified as Typic Hapludult and Typic Hapludox [64].

**Figure 10:**
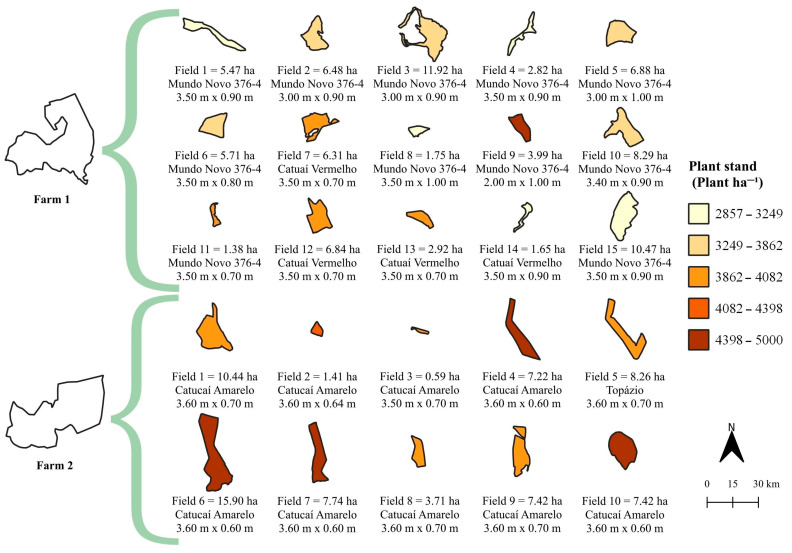
Area (ha), variety, plant spacing and plant stand of the plots, considered as cell, from coffee farms 1 and 2. For each cell, note: Field n = Area (ha); coffee variety; row spacing (m) × plant spacing (m).

Farm 1 (production area of 82.88 ha, 21°59′46″ S, 45°52′51″ W, altitude de 877.00 m) is composed of 15 plots, considered as cell, with “Mundo Novo 376-4” and “Catuaí Vermelho” varieties. Farm 2 (production area of 72.12 ha, 21°57′8″ S, 45°51′45″ W, altitude of 950.00 m) is composed of 10 cells cultivated with “Catucaí Amarelo” and “Topázio” varieties, totaling 25 cells in the farms (Figure 10). The design of the study (size of the plots—cells) was based on the topography (relief) and other farmer’s criteria related to the technological level of the farm.

On both farms, coffee plantations were ten years old in 2018 and were conducted under conventional management practices (application of herbicide, mineral fertilization of N-P-K, foliar application of fungicide and insecticide, pruning and mowing of weeds between plant rows), without irrigation system and using a mechanized harvesting system. Farms have characteristics typical of the Southern mesoregion of Minas Gerais in terms of size (predominance of small coffee-growing areas), coffee varieties and management methods (e.g., no use of irrigation equipment), as well as little or no mechanized cultivation in areas of sloping topography [65], largely reflecting the practices adopted by local producers.

**Figure 11:**
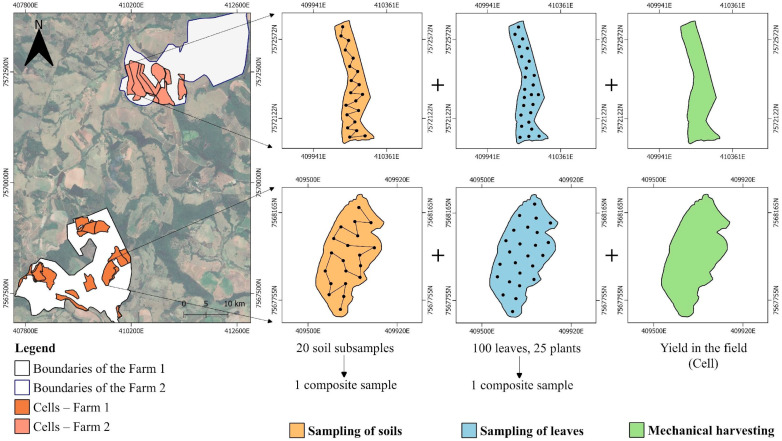
Cell-size strategy for soil and plant sampling.

Dataset was composed of yield and soil attributes from 2018, 2019, 2020 and 2021. In addition, the nutrient leaf content of the coffee plants was examined for the year 2020. Average value of each variable was attributed to each cell (Figure 11). Yield data were obtained based on the wet mass and total volume per cell. For each cell, ten samples of 2.00 L were dried to obtain the conversion factor to kg and then the total volume was converted into Mg ha^−1^ after grain processing.

For each cell, a composite soil sample was collected with 20 subsamples randomly distributed at 0–0.20 m depth, and macro- and micronutrients were analyzed. Most of the coffee plant root hairs are concentrated in the upper soil layers, between 0 and 0.30 m, which makes the quality of the topsoil more relevant to coffee plants than deeper subsoil [66]. Chemical attributes of the soil analyzed were pH, using soil in water (1:2.5), exchangeable calcium (Ca), magnesium (Mg), aluminum (Al) and potassium (K), potential acidity (H + Al), total cation exchange capacity (CEC), remaining phosphorus (P rem), assimilable phosphorus (P), sulphur (S), organic matter (OM) and available micronutrient content, manganese (Mn), iron (Fe), copper (Cu) and zinc (Zn), according to Teixeira et al. [67]. Available boron (B) in the soil was analyzed using the hot water extractor [68]. Average values of the soil attributes in the years 2018–2021 for farms 1 and 2 can be seen in Table 2. Leaf sampling was conducted gathering the third and fourth pair of leaves at the middle third of the plants from the plagiotropic branch at the cardinal points free of disease, injuries and pest. Leaves were stored in paper bag and sent to macro- (Ca, K, Mg, nitrogen—N, P and S) and micronutrient (B, Cu, Fe, Mn and Zn) analysis according to Malavolta et al. [69].

The biennial effect is evaluated in pairs and calculated by subtracting the average between high and low productive years according to Equation (1) [70]:

(1) $${MB}_{cell}=\frac{\left(P_{1}+P_{2}\right)}{2}-\frac{\left(P_{3}+P_{4}\right)}{2}$$

where *MB_cell_* = magnitude of bienniality; *P*_1_, *P*_2_ = average yield value from the highest productive years, Mg ha^−1^; *P*_3_, *P*_4_ = average yield value from the lowest productive years, Mg ha^−1^.

### 3.2. Optimizing Soil Fertilization

Phosphate (P_2_O_5_) and potassium (K_2_O) soil fertilization recommendations were obtained for subsequent harvests on farms 1 and 2, covering the years 2019, 2020, 2021 and 2022 (e.g., soil analysis and yield data from 2018 were used for 2019 and so on). N data were not available, except for 2021, so N recommendations were not obtained. Fertilizer doses were obtained in two strategies for each cell: (i) using the average levels of soil attributes and yield of the farm (Uniform Management—UM) and (ii) assigning the levels of soil attributes and yield specific to the respective cells (Localized Management—LM_cell_). In this way, soil fertilization recommendations were extracted based on the farm’s average data and those optimized by the cell sampling method.

Recommendations were made considering the nutrient exportation based on yield data. Considering the total need of the coffee tree (vegetation and production), for each 60 kg of processed coffee, 0.60 kg of P_2_O_5_ and 5.90 kg of K_2_O are required per hectare [71]. For farms or cells that did not show yield values in a given year (yield equal to zero) due to skeletonization—which involves halting the production of vegetative branches in the pruning year to ensure the reestablishment of productive branches to the following year [57,72]—the recommendation for P and K fertilization was made based on the availability of these nutrients in the soil and expected yield, using historical yield data for that farm or cell [71,73].

### 3.3. Economic Analysis

Profitability analysis was carried out for farm 1 for the years 2018 to 2021. Economic analysis was carried out considering the following costs, provided by the farm’s financial manager: (A) Effective operating cost. This includes administration costs, crop management costs (related to certain activities and management, such as pruning, including skeletonization), management costs (related to the management of the farm, such as technical assistance to monitor the crop and electricity), soil fertilization costs (which include the costs of labor and equipment for soil sampling, laboratory soil analysis, inputs and fertilization operations), leaf fertilization (labor and equipment for leaf sampling, leaf analysis, inputs and fertilization operation), pest and disease control (such as integrated pest management—IPM), weed control, harvesting (related to the entire cost of mechanized harvesting), post-harvesting (related to activities after the grain has been harvested, such as costs of mechanical dryers, storage and transport of the grain for sale) and marketing. (B) Total operating cost, corresponding to the effective operating cost (A) and depreciation of installations, machines and implements. (C) Total cost, corresponding to the sum of the return on capital and the total operating cost (B) (summarized data, Table 3, and complete data, Supplementary Material S2, Table S1).

Average price of the coffee was retrieved by year (2018–2021) according to Centro de Estudos Avançados em Economia Aplicada (in Portuguese) (CEPEA-Esalq/USP) [74]. The conversion of Brazilian currency (BRL) to United States dollar currency (USD) was based on the average annual data from the “Instituto de Pesquisa Econômica Aplicada” (in Portuguese) (IPEA) [75]. The exchange rate from BRL to USD for 2018, 2019, 2020 and 2021 was 3.6542, 3.9451, 5.1558 and 5.3950, respectively. Profitability was calculated subtracting the total income by the total production cost.

Subsequently, the total P_2_O_5_ and K_2_O recommended for application on farms 1 and 2 from 2019 to 2022 were calculated. From this, the percentage (%) difference in total inputs between the LM_cell_ strategy and UM for each year was obtained. % values equal to zero indicate that the total amount of fertilizer recommended by LM_cell_ and UM are similar; positive values indicate that the LM_cell_ strategy presented higher recommendation values than UM, and negative values indicate the opposite, providing input savings.

### 3.4. Statistical Analysis and Surface Mapping

Descriptive analysis (minimum, average and maximum values, coefficient of variance—CV and standard deviation—Supplementary Material S3, Tables S2 and S3) was carried out on the virtual environment JupyterLab using the Python v. 3.10.5 programming language [76,77]. Pearson’s correlation analysis was applied considering *p*-value lower than 0.05. Surface maps were created using the geographic information system QGIS v. 3.22.1 [78].

## 4. Conclusions

In the absence of resources for high-resolution monitoring, the cell-level approach is able to provide important data to the coffee farmer considering the spatio-temporal variability. Soil and plant attributes, including yield, biennial effect and profitability maps, were generated at the cell level and showed cell heterogeneity among them. Thus, customize cell-level management could be applied, improving coffee farmers’ sustainability.

The final considerations of this study are as follows:

1. The cell-size sampling strategy identified high- and low-yielding regions over time, associating them with their biennial effect. Consequently, the identification of profitable areas can guide the management of resources and inputs in the subsequent harvest.
2. The cell-size approach optimized the recommendation of potassium and phosphate fertilizers on farms, demonstrating that localized management can be carried out regardless of the spatial resolution data.
3. Although this method was applied to coffee cultivation, cell sampling indicates that it is a strategy applicable to other crops and regions in various contexts.
4. This methodology can be applied in scenarios with limited resources for high-density sampling, especially small- and medium-sized farmers, integrating them into the adoption of precision agriculture practices.

## Figures and Tables

**Table 1:** Total phosphate (P_2_O_5_) and potassium (K_2_O) fertilization recommended for application on farms 1 and 2 from 2019 to 2022.

Farm	Fertilizer	Management	2019	2020	2021	2022
1	P_2_O_5_	UM (kg)	2575.85	1491.79	1720.06	888.08
		LM_cell_ (kg)	2604.56	1345.39	1793.09	1578.56
		Difference (kg)	28.71	−146.40	73.03	690.49
		%	1.11	−9.81	4.25	77.75
	K_2_O	UM (kg)	25,329.19	24,863.10	16,913.95	8732.76
		LM_cell_ (kg)	24,931.94	14,037.45	17,632.04	13,455.99
		Difference (kg)	−397.25	−10,825.65	718.09	4723.24
		%	−1.57	−43.54	4.25	54.09
2	P_2_O_5_	UM (kg)	1700.50	1803.00	1477.91	0.00
		LM_cell_ (kg)	1841.42	1575.37	1541.26	1228.76
		Difference (kg)	140.92	−227.63	63.35	1228.76
		%	8.29	−12.63	4.29	100.00
	K_2_O	UM (kg)	16,721.61	16,227.00	14,532.80	16,227.00
		LM_cell_ (kg)	18,107.33	12,410.81	15,155.73	16,292.85
		Difference (kg)	1385.72	−3816.19	622.93	65.85
		%	8.29	−23.52	4.29	0.41

UM: Uniform Management; LM_cell_: localized management in cell-size resolution; %: percentage difference in total inputs for the LM_cell_ strategy compared to the UM; values equal to 0.00: no fertilization.

**Table 2:** Average values of the soil results obtained from 2018 to 2021 for farms 1 and 2.

Variables	Unit	Farm 1				Farm 2			
		2018	2019	2020	2021	2018	2019	2020	2021
pH H_2_O	-	5.47	5.67	5.03	5.62	5.07	5.67	5.41	5.43
P rem	mg dm^−3^	20.02	21.12	23.89	20.81	14.44	16.29	22.10	15.39
P	mg dm^−3^	3.01	17.29	9.71	8.16	3.43	7.19	1.50	13.09
K	mg dm^−3^	63.47	89.53	73.00	78.60	77.30	98.65	63.00	101.90
Ca	cmol_c_ dm^−3^	3.02	2.87	2.09	3.83	1.39	2.10	1.97	2.92
Mg	cmol_c_ dm^−3^	0.84	0.74	0.52	0.83	0.37	0.59	0.54	0.76
H + Al	cmol_c_ dm^−3^	3.83	3.32	4.89	3.53	5.00	3.45	3.21	4.12
CEC	cmol_c_ dm^−3^	7.94	7.08	7.68	8.36	6.96	6.43	5.88	8.05
OM	g kg^−1^	30.60	31.20	16.70	22.10	28.10	31.90	17.70	21.30
S	mg dm^−3^	4.22	6.60	4.98	5.07	4.13	3.57	3.58	4.62
Mn	mg dm^−3^	17.18	19.79	11.73	17.37	8.33	8.54	5.51	10.25
Fe	mg dm^−3^	53.03	58.47	62.43	64.79	55.97	41.52	42.49	66.10
Cu	mg dm^−3^	0.77	1.18	1.08	0.93	1.19	0.94	1.00	1.64
Zn	mg dm^−3^	1.36	1.79	2.56	1.98	1.36	2.90	0.94	3.04
B	mg dm^−3^	0.47	0.69	0.34	0.45	0.76	0.57	0.39	0.36

pH H_2_O: pH using soil in water (1:2.5); P rem: remaining Phosphorus (mg dm^−3^); P: Phosphorus (mg dm^−3^); K: Potassium (mg dm^−3^); Ca: Calcium (cmol_c_ dm^−3^); Mg: Magnesium (cmol_c_ dm^−3^); H + Al: Potential acidity (cmol_c_ dm^−3^); CEC: Cation Exchange Capacity (cmol_c_ dm^−3^); OM: Organic Matter content (g kg^−1^); S: Sulphur (mg dm^−3^); Mn: Manganese (mg dm^−3^); Fe: Iron (mg dm^−3^); Cu: Cooper (mg dm^−3^); Zn: Zinc (mg dm^−3^); B: Boron (mg dm^−3^).

**Table 3:** Coffee production cost, in USD ha^−1^, from 2018 to 2021 for farm 1.

Variables	Year			
	2018	2019	2020	2021
Effective operating cost (US$ ha^−1^)	2583	2393	1831	1750
Total operating cost (US$ ha^−1^)	3266	3025	2315	2212
Total cost (US$ ha^−1^)	3637	3369	2577	2463
Average coffee price (US$ bag^−1^) *	120	107	105	178

* Average coffee price from CEPEA-Esalq/USP [59]. One bag corresponds to 60.00 kg of processed coffee.

## Data Availability

The original contributions presented in this study are included in the article/Supplementary Materials; further inquiries can be directed to the corresponding author.

## Supplementary Materials

The following supporting information can be downloaded at: https://www.mdpi.com/article/10.3390/plants14020169/s1, Supplementary Material S1 (Figure S1: Recommended doses of phosphate (P_2_O_5_) (a) and potassium (K_2_O) (b) fertilizers by the uniform management (UM—red dashed line) and localized management at cell resolution (LM_cell_—blue and grey bar chart) strategies for farm 1 for 2019 to 2022; Figure S2: Recommended doses of phosphate (P_2_O_5_) (a) and potassium (K_2_O) (b) fertilizers by the uniform management (UM—red dashed line) and localized management at cell resolution (LM_cell_—blue and grey bar chart) strategies for farm 2 for 2019 to 2022); Supplementary Material S2 (Table S1: Details of production costs from 2018 to 2021 for farm 1); Supplementary Material S3 (Table S2: Descriptive analysis of yield and soil attributes for farm 1 in 2018 to 2021; Table S3: Descriptive analysis of yield and soil attributes for farm 2 in 2018 to 2021).
