# Supplementary material for: Spatial and Temporal Variability Management for All Farmers: A Cell-Size Approach to Enhance Coffee Yields and Optimize Inputs
Source: Plants (Basel). 2025 Jan 9;14(2):169. doi: 10.3390/plants14020169 (PMC11769169; doi:10.3390/plants14020169)
Supplement: Supplementary file 1 [file plants-14-00169-s001.zip › plants-3362059-supplementary.pdf]

Title: Spatial and Temporal Variability Management for All Farmers: A Cell-Size Approach to Enhance Coffee Yields and Optimize Inputs

Journal: Plants

Author names: Eudocio Rafael Otavio da Silva, Thiago Lima da Silva, Marcelo Chan Fu Wei, Ricardo Augusto de Souza, José Paulo Molin

Affiliation and e-mail address: University of São Paulo, Luiz de Queiroz College of Agriculture, Department of Biosystems Engineering, Laboratory of Precision Agriculture (LAP), Piracicaba, 13418-900, São Paulo, Brazil, eudocio@usp.br

Supplementary Material S1

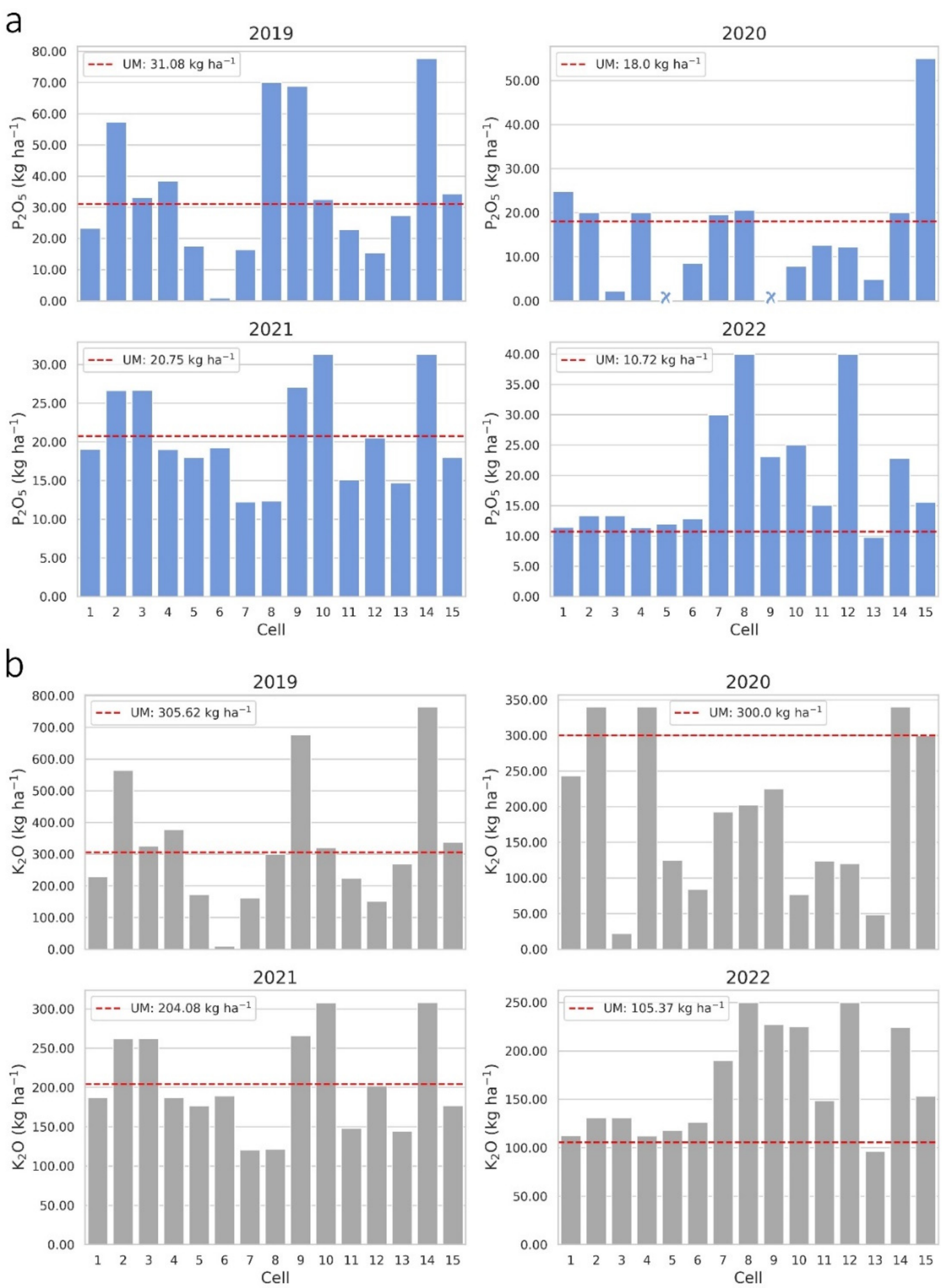

Figure S1. Recommended doses of phosphate (P_2O_5) (a) and potassium (K_2O) (b) fertilizers by the uniform management (UM – red dashed line) and localized management at cell resolution (LM_{cell} – blue and grey bar chart) strategies for farm

1 for 2019 to 2022. The blue x on the axis corresponding to the value zero indicates that the recommendation is not to fertilize using the LM_{cell} strategy.

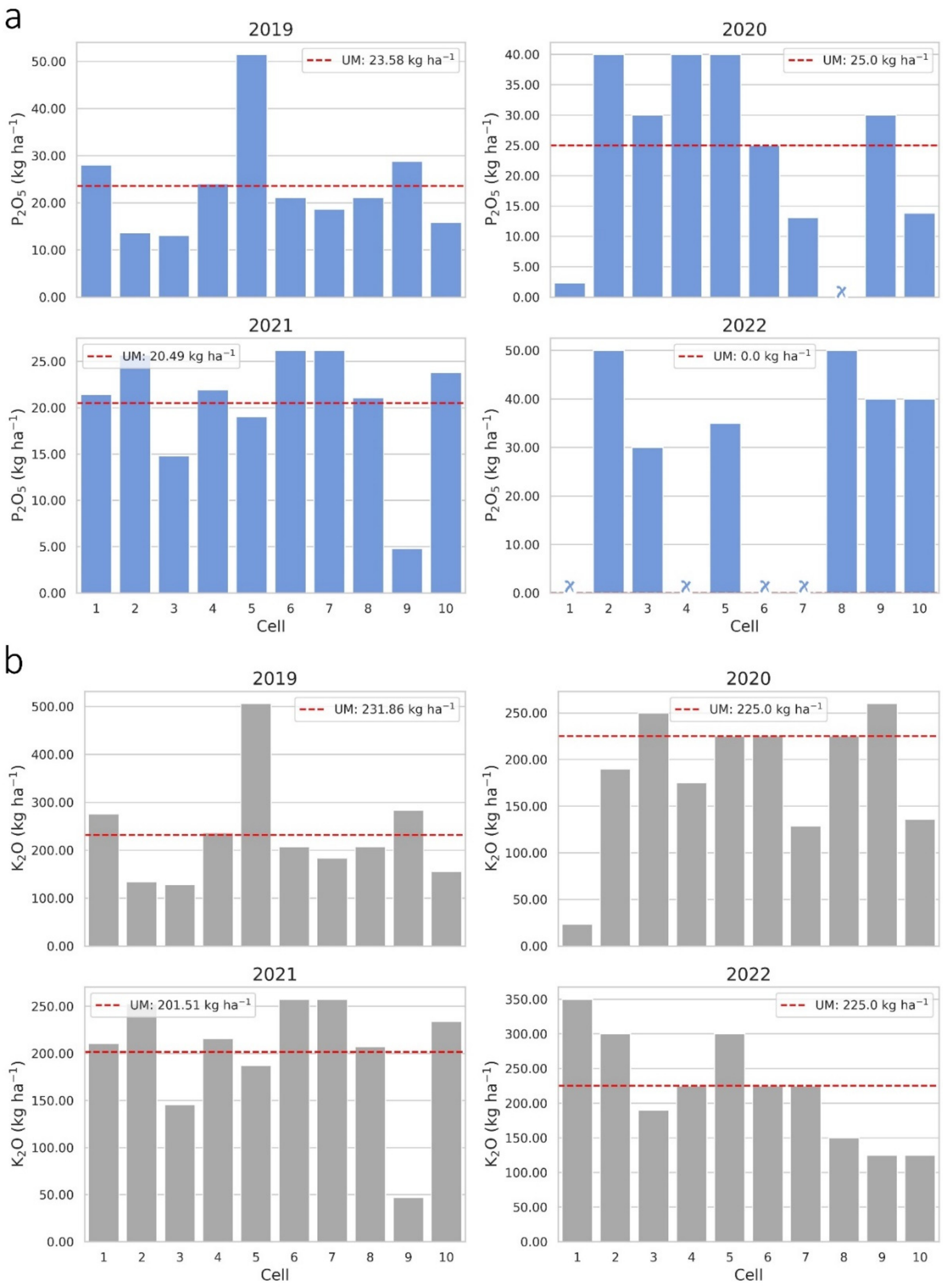

Figure S2. Recommended doses of phosphate (P_2O_5) (a) and potassium (K_2O) (b) fertilizers by the uniform management (UM – red dashed line) and localized management at cell resolution (LM_{cell} – blue and grey bar chart) strategies for farm 2 for 2019 to 2022. The blue x on the axis corresponding to the value zero indicates that the recommendation is not to fertilize using the LM_{cell} strategy. UM: 0.0 kg ha^{-1} corresponds to the recommendation not to fertilize.

Supplementary Material S2

Table S1. Details of production costs from 2018 to 2021 for farm 1.

Variables	Year			
	2018	2019	2020	2021
**Effective operating cost (U$ ha^{-1})**	2,583.84	2,393.32	1,831.31	1,750.11
Administration (US$ ha^{-1})	342.70	317.43	242.89	232.12
Fertilization via leaves (US$ ha^{-1})	48.68	45.09	34.50	32.97
Fertilization via soil (US$ ha^{-1})	597.90	553.81	423.77	404.98
Harvest (US$ ha^{-1})	537.69	498.04	381.09	364.19
Commercialization (US$ ha^{-1})	204.63	189.54	145.03	138.60
Crop management (US$ ha^{-1})	30.99	28.71	21.97	20.99
Weed control (US$ ha^{-1})	180.52	167.20	127.94	122.27
Pest and disease control (US$ ha^{-1})	385.85	357.40	273.48	261.35
Management (US$ ha^{-1})	42.17	39.06	29.89	28.56
Post-harvest (US$ ha^{-1})	212.70	197.01	150.75	144.07
**Total operating cost (US$ ha^{-1})**	3,266.48	3,025.62	2,315.13	2,212.49
Effective Operating Cost (US$ ha^{-1})	2,583.84	2,393.32	1,831.31	1,750.11
Depreciation (US$ ha^{-1})	682.64	632.30	483.82	462.37
**Total cost (US$ ha^{-1})**	3,637.31	3,369.10	2,577.96	2,463.66
Total Operating Cost (US$ ha^{-1})	3,266.48	3,025.62	2,315.13	2,212.49
Return on Capital (US$ ha^{-1})	370.83	343.49	262.83	251.18
Average coffee price (US$ bag^{-1})*	120.82	107.56	105.74	177.92

* Average coffee price from CECAFE (2024).

Supplementary Material S3

Table S2. Descriptive analysis of yield and soil attributes for farm 1 in 2018 to 2021.

Year	Variable	Yield	pH	P rem	P	K	Ca	Mg	H + Al	CEC	OM	S	Mn	Fe	Cu	Zn	B
2018	Mean	3.11	5.47	20.02	3.01	63.47	3.02	0.84	3.83	7.94	30.60	4.22	17.18	53.03	0.77	1.36	0.47
	Min	0.00	4.82	10.33	1.46	42.00	1.15	0.41	1.75	5.16	18.70	2.30	6.80	23.90	0.30	0.70	0.20
	Max	7.77	6.42	26.71	4.95	88.00	6.12	1.83	6.79	10.89	46.00	10.50	37.20	134.60	2.00	2.20	0.90
	std	2.24	0.44	4.84	1.13	16.51	1.49	0.41	1.30	1.77	1.00	2.00	8.87	30.88	0.52	0.43	0.21
	CV	72.04	7.97	24.19	37.45	26.02	49.46	49.00	34.01	22.29	32.58	47.47	51.65	58.23	67.72	31.29	45.58
2019	Mean	0.76	5.67	21.12	17.29	89.53	2.87	0.74	3.32	7.08	31.20	6.60	19.79	58.47	1.18	1.79	0.69
	Min	0.00	4.85	7.83	6.73	56.00	0.71	0.30	2.05	6.02	14.10	3.50	3.10	19.20	0.30	0.60	0.50
	Max	2.48	6.59	31.68	61.68	159.00	5.24	1.33	4.90	9.30	42.90	14.60	32.80	128.90	3.00	9.60	1.20
	std	0.86	0.45	6.02	17.99	31.82	1.23	0.29	0.93	0.87	0.80	3.29	7.98	33.09	0.91	2.26	0.18
	CV	100.00	7.97	28.49	100.00	35.54	42.72	39.72	27.93	12.29	25.52	49.83	40.33	56.60	77.02	100.00	26.89
2020	Mean	2.08	5.03	23.89	9.71	73.00	2.09	0.52	4.89	7.68	16.70	4.98	11.73	62.43	1.08	2.56	0.34
	Min	1.22	4.38	10.78	0.95	34.00	0.70	0.20	3.18	6.04	6.50	3.50	4.10	22.60	0.40	0.40	0.20
	Max	3.14	5.46	33.04	27.34	137.00	3.52	0.84	7.55	9.51	27.40	6.90	30.80	159.50	3.00	8.30	0.50
	std	0.64	0.32	6.00	7.28	27.22	0.78	0.18	1.21	1.10	0.53	0.98	6.20	39.81	0.85	2.31	0.10
	CV	30.75	6.36	25.13	75.02	37.29	37.21	34.95	24.77	14.36	31.41	19.65	52.91	63.76	78.74	90.31	28.99
2021	Mean	1.07	5.62	20.81	8.16	78.60	3.83	0.83	3.53	8.36	22.10	5.07	17.37	64.79	0.93	1.98	0.45
	Min	0.00	4.95	11.13	1.97	51.67	1.47	0.37	1.39	6.33	9.70	2.90	10.20	30.87	0.37	0.87	0.20
	Max	2.31	6.53	28.73	31.17	136.00	7.25	1.48	5.81	12.46	33.70	8.27	24.80	141.10	2.40	5.80	0.90
	std	0.77	0.46	5.47	8.11	20.58	1.74	0.34	1.41	1.54	0.68	1.55	4.71	34.51	0.59	1.34	0.22
	CV	71.58	8.25	26.28	99.31	26.19	45.48	40.78	40.14	18.40	30.85	30.52	27.09	53.27	63.55	67.60	48.06

Min: Minimum (Mg ha^{-1}); Max: Maximum (Mg ha^{-1}); std: standard deviation; CV: coeficiente of variation (%); yield (Mg ha^{-1}); pH: pH using soil in water (1:2.5); P rem: remaining Phosphorus (mg dm^{-3}); P: Phosphorus (mg dm^{-3}); K: Potassium (mg dm^{-3}); Ca: Calcium (cmol$_c$ dm^{-3}); Mg: Magnesium (cmol$_c$ dm^{-3}); H+Al: Potential acidity (cmol$_c$ dm^{-3}); CEC: Cation Exchange Capacity (cmol$_c$ dm^{-3}); OM: Organic Matter content (g kg^{-1}); S: Sulphur (mg dm^{-3}); Mn: Manganese (mg dm^{-3}); Fe: Iron (mg dm^{-3}); Cu: Cooper (mg dm^{-3}); Zn: Zinc (mg dm^{-3}); B: Boron (mg dm^{-3}).

Table S3. Descriptive analysis of yield and soil attributes for farm 2 in 2018 to 2021.

Year	Variable	Yield	pH	P rem	P	K	Ca	Mg	H + Al	CEC	OM	S	Mn	Fe	Cu	Zn	B
	Mean	2.36	5.07	14.44	3.43	77.30	1.39	0.37	5.00	6.96	28.10	4.13	8.33	55.97	1.19	1.36	0.76
	Min	1.31	4.65	11.06	1.52	57.00	0.54	0.16	3.85	5.67	18.70	2.60	2.30	34.10	0.70	0.40	0.30
2018	Max	5.15	5.25	21.83	6.43	100.00	1.85	0.52	7.39	8.35	38.40	5.70	31.30	121.80	1.70	2.50	1.10
	std	1.12	0.19	3.98	1.51	14.06	0.45	0.12	1.21	0.80	0.65	0.95	8.65	27.23	0.29	0.69	0.28
	CV	47.58	3.74	27.57	43.96	18.19	32.18	31.35	24.31	11.53	23.17	23.11	103.90	48.65	24.24	50.97	37.32
	Mean	0.29	5.67	16.29	7.19	98.65	2.10	0.59	3.45	6.43	31.90	3.57	8.54	41.52	0.94	2.90	0.57
	Min	0.00	4.75	14.32	2.01	53.00	0.97	0.21	1.41	5.00	24.80	2.00	2.30	22.90	0.50	1.10	0.40
2019	Max	1.38	6.54	19.48	16.06	191.00	3.50	1.01	5.62	7.58	44.50	7.60	24.20	66.20	1.50	4.40	0.70
	std	0.56	0.52	1.69	4.66	41.94	0.78	0.31	1.35	0.82	0.61	1.55	6.22	13.81	0.33	0.96	0.10
	CV	191.03	9.22	10.36	64.81	42.51	37.37	53.05	39.11	12.72	19.03	43.32	72.86	33.26	35.50	33.18	18.07
	Mean	2.05	5.41	22.10	1.50	63.00	1.97	0.54	3.21	5.88	17.70	3.58	5.51	42.49	1.00	0.94	0.39
	Min	0.48	4.81	17.14	0.95	47.00	0.74	0.28	2.09	4.73	14.10	2.70	1.00	24.00	0.50	0.40	0.20
2020	Max	2.62	5.75	30.97	2.60	75.00	3.36	0.84	5.33	7.02	26.10	4.70	8.90	67.60	1.50	1.70	0.60
	std	0.66	0.34	4.30	0.55	9.68	0.80	0.17	1.04	0.68	0.38	0.56	3.06	12.75	0.33	0.50	0.12
	CV	32.01	6.33	19.46	36.65	15.37	40.49	30.82	32.57	11.57	21.68	15.74	55.51	30.00	32.69	52.90	30.85
	Mean	-	5.43	15.39	13.09	101.90	2.92	0.75	4.12	8.05	21.30	4.62	10.25	66.10	1.64	3.04	0.36
	Min	-	4.58	9.51	1.66	29.00	0.83	0.35	2.42	6.17	17.50	2.50	3.20	36.30	0.90	0.90	0.20
2021	Max	-	5.80	24.81	49.31	146.00	4.23	1.04	8.75	10.63	28.70	7.60	27.50	110.00	2.40	12.10	0.60
	std	-	0.38	4.50	16.11	36.62	1.09	0.24	1.97	1.55	0.36	1.64	7.12	21.71	0.48	3.30	0.15
	CV	-	6.93	29.28	123.07	35.94	37.22	32.27	47.87	19.24	16.78	35.42	69.43	32.85	29.06	108.66	41.82

Min: Minimum (Mg ha^{-1}); Max: Maximum (Mg ha^{-1}); std: standard deviation; CV: coeficiente of variation (%); yield (Mg ha^{-1}); pH: pH using soil in water (1:2.5); P rem: remaining Phosphorus (mg dm^{-3}); P: Phosphorus (mg dm^{-3}); K: Potassium (mg dm^{-3}); Ca: Calcium (cmol$_c$ dm^{-3}); Mg: Magnesium (cmol$_c$ dm^{-3}); H+Al: Potential acidity (cmol$_c$ dm^{-3}); CEC: Cation Exchange Capacity (cmol$_c$ dm^{-3}); OM: Organic Matter content (g kg^{-1}); S: Sulphur (mg dm^{-3}); Mn: Manganese (mg dm^{-3}); Fe: Iron (mg dm^{-3}); Cu: Cooper (mg dm^{-3}); Zn: Zinc (mg dm^{-3}); B: Boron (mg dm^{-3}).
